# The adipose-derived mesenchymal stem cell secretome promotes hepatic regeneration in miniature pigs after liver ischaemia-reperfusion combined with partial resection

**DOI:** 10.1186/s13287-021-02284-y

**Published:** 2021-03-30

**Authors:** Zhihui Jiao, Yajun Ma, Qianzhen Zhang, Yue Wang, Tao Liu, Xiaoning Liu, Chenxi Piao, Boyang Liu, Hongbin Wang

**Affiliations:** 1 College of Veterinary Medicine, Northeast Agricultural University, Harbin, 150030 People’s Republic of China; 2 College of Wildlife and Protected Area, Northeast Forestry University, Harbin, People’s Republic of China

**Keywords:** ASC-secretome, Hepatic regeneration, Inflammatory response, Ischaemia-reperfusion, Miniature pig

## Abstract

**Background:**

Hepatic ischaemia-reperfusion injury (HIRI) is inevitable in complicated liver surgery and is a major factor leading to postoperative complications and liver dysfunction. Studies have shown that the paracrine mechanisms of stem cell may be essential to tissue repair and functional improvement after transplantation. However, the role of the adipose-derived mesenchymal stem cell secretome (ASC-secretome) in liver regeneration in large animals remains to be determined.

**Methods:**

Twenty-four miniature pigs were subjected to laparoscopic liver ischaemia-reperfusion combined with partial hepatectomy and divided into the following four groups: the saline group, the DMEM group, the ASC group and the ASC-secretome group. Serum and liver tissue samples were collected before the operation and at 1, 3 and 7 days after the operation, and changes in tissue pathology, serum inflammation, liver function, angiogenesis-related factors and liver tissue regeneration-related genes and proteins were evaluated.

**Results:**

Detailed histological analysis showed that ASCs and the ASC-secretome changed pathological damage to liver tissue after liver ischaemia-reperfusion combined with partial hepatectomy (1 and 3 days: *p* < 0.01). Compared with the saline and DMEM control groups, the ASC-secretome group had significantly reduced expression levels of ALP (1 and 3 days: *p* < 0.05), ALT (1 day: *p* < 0.01; 3 days: *p* < 0.05) and AST (1 and 3 days: *p* < 0.01), which promoted the recovery of liver function. Moreover, detection of the expression levels of TNF-α and IL-1β (1 day: *p* < 0.01; 3 days: *p* < 0.05), IL-6 (1 and 3 days: *p* < 0.05) and IL-10 (1 and 3 days: *p* < 0.01) in serum confirmed that the ASC-secretome had obvious anti-inflammatory effects. In addition, the ASC-secretome increased the expression levels of ANG-1 (3 days: *p* < 0.01), ANG-2 (3 and 7 days: *p* < 0.01) and VEGF (1 and 7 days: *p* < 0.05; 3 days: *p* < 0.01) and promoted angiogenesis during liver regeneration. Moreover, it promoted the mRNA expression of HGF and Cyclin D1 (1 and 3 days: *p* < 0.01); increased the levels of p-STAT3 (1 and 3 days: *p *< 0.01), PCNA and Ki67 (1 and 3 days: *p* < 0.01; 7 days: *p* < 0.05); inhibited the negative feedback of SOCS3 (1 and 3 days: *p* < 0.01); and decreased the mRNA expression of TGF-β (3 days: *p* < 0.01). The cytokines and growth factors detected in the ASC-secretome included TNF-α, IL-6, IL-1β, ANG-1, ANG-2, VEGF and b-FGF.

**Conclusion:**

The ASC-secretome alleviates the inflammatory response induced by ischaemia-reperfusion combined with partial hepatectomy in miniature pigs and promotes liver regeneration.

## Background

Ischaemia-reperfusion injury is a pathological phenomenon in which tissue and cell damage in hypoxic organs increases after the oxygen supply is restored [1]. Complex liver surgery inevitably leads to hepatic ischaemia-reperfusion injury (HIRI), which is a key factor leading to postoperative complications and liver dysfunction [2]. The most effective treatment for end-stage liver disease is orthotopic liver transplantation (OLT). However, its application is limited due to the shortage of organ donors, the high cost and immunosuppression [3, 4].

The use of stem cell therapy as a treatment method for liver diseases has been studied in more than 500 clinical trials for several inflammatory diseases and liver diseases [5]. The mechanism by which stem cells exert their therapeutic effects is not yet fully understood. Studies have shown that the paracrine mechanism by which stem cells release soluble cytokines may be essential for tissue repair and functional improvement after cell transplantation [6]. Mesenchymal stem cell-conditioned medium (MSC-CM) is considered the next generation of regenerative medicine [7]. Preclinical studies have shown that the use of MSC-CM can reduce liver damage [8]. Some studies have even proven that direct injection of factors secreted by stem cells can have a better therapeutic effect than transplantation of the cells themselves [9]. This is mainly because a variety of nutritional factors secreted by stem cells can suppress immune and inflammatory responses, thereby promoting the repair and regeneration of damaged tissues. Cell-free therapy provides a promising method for future liver regenerative medicine.

The adipose-derived mesenchymal stem cell secretome (ASC-secretome) is abundant and easily stored and can be produced rapidly. The MSC secretome is effective in treating myocardial infarction [10], psoriasis vulgaris [11] and osteoarthritis [12], regulating glucose levels [13], and attenuating sepsis [14], among other effects. It has been shown that the MSC secretome in culture medium, known as conditioned medium, contains a variety of components, such as cytokines, growth factors, extracellular matrix proteins, extracellular matrix proteases, and hormones [15, 16]. To date, most studies on stem cell-conditioned medium in liver regeneration have been in rodents [17, 18], but the role of the ASC-secretome in liver regeneration in large animals remains to be determined. At present, there is no report discussing the role of the ASC-secretome in liver regeneration in miniature pigs after liver ischaemia-reperfusion combined with partial resection. We established animal models through laparoscopic technology and evaluated the therapeutic potential of the ASC-secretome by transplanting ASCs and the ASC-secretome. The results provide evidence that the ASC-secretome can relieve inflammation and promote liver regeneration.

## Materials and methods

### Animals

The study was performed on 4- to 6-month-old miniature pigs with body weights of 20–25 kg. Animal housing and experiments were complied with the Institutional Animal Care and Use Committee of Northeast Agricultural University.

### Production of CM containing the secretion products of ASCs

ASCs were isolated from the abdominal subcutaneous fat tissue of miniature pigs. The isolation and culture of ASCs were carried out according to a method described previously [19]. In short, adipose tissue was subjected to type I collagenase digestion, centrifugation, filtration and other steps to ultimately yield ASCs. Before producing the CM, fourth-generation 80% confluent ASCs were thoroughly washed with phosphate-buffered saline to remove serum. Then, Dulbecco’s modified Eagle’s medium with low glucose was added. The ASCs were cultured under serum-free starvation conditions for 48 h, and then the CM was collected and purified by centrifugation at 3000 g for 10 min. Next, 12 mL of purified CM was collected into a 3-kDa ultrafiltration unit (Millipore, Billerica, USA) and centrifuged at 5000*g* for 1.5 h at 4 °C. Finally, approximately 500 μL of supernatant, which is hereafter referred to as the “ASC-secretome”, was collected from every ultrafiltration unit. Then, the ASC secretome was stored at − 80 °C for subsequent experiments.

### Establishment of HIRI in miniature pigs and infusion of ASCs or ASC-secretome

The experimental animals were fed a piglet diet (Jinxinnong Feed, Shenzhen, China) and tap water and were housed at a stable temperature (20 °C) with a 12-h light-dark cycle. The surgery was performed after 12 h of fasting and 2 h of water restriction. The miniature pigs were anaesthetized by isoflurane inhalation for laparoscopic surgery. First, the right portion of the liver was subjected to ischaemia for 60 min, and then the left portion of the liver was subjected to hepatectomy after the right portion of the liver was perfused. All substances were administered to miniature pigs through the liver parenchyma immediately after partial hepatectomy. According to the substances used, the twenty-four miniature pigs were divided into the following four groups, with six animals in each group: the saline group, the DMEM group, the ASC group and the ASC-secretome group. The saline and DMEM groups were used as the control groups for the ASC and ASC-secretome groups, respectively; and the ASC and ASC-secretome groups were the treatment groups. After hepatectomy, individual miniature pigs were administered either normal saline (saline group), L-DMEM (DMEM group), ASCs (ASC group) or ASC-secretome (ASC-secretome group). The number of transplanted ASCs was not fixed and depending on the weight of the miniature pig (1 × 10^6^ cells/kg). Similarly, the volume of the transplanted ASC-secretome was determined according to the number of cells depending on the body weight of the animal. In this way, the treatment groups were administered ASCs or an equivalent amount of ASC-secretome. At specific time points after surgery (1, 3 and 7 days), laparoscopic minimally invasive techniques were used to collect liver tissue specimens for subsequent analysis. All animals were given Tolfedine 4% (Vetoquinol SA, France) for analgesia and survived well after surgery.

### Histological analysis

For histopathological analysis, liver tissue was fixed with 10% formalin, processed and embedded in paraffin. Serial sections (5 μm thick) were stained with haematoxylin and eosin. We assessed the severity of liver damage based on the previously reported Suzuki classification system [20], which includes the following parameters with scores of 0 to 4: parenchymal necrosis, sinusoidal congestion and cytoplasmic vacuolization. A professional pathologist examined three liver slices from each pig and scored three randomly selected fields of view from each liver slice. Then, the average score of each animal was determined by the sum of all scores.

### Analysis of blood samples

Serum was collected from miniature pig blood samples centrifuged at 3500 rpm for 15 min. Alkaline phosphatase (ALP), alanine aminotransferase (ALT), aspartate aminotransferase (AST), angiopoietin-1 (ANG-1), angiopoietin-2 (ANG-2), vascular endothelial growth factor (VEGF), tumour necrosis factor (TNF-α), interleukin-6 (IL-6), interleukin-1β (IL-1β) and interleukin-10 (IL-10) were investigated using commercial kits (Nanjing Jiancheng Bioengineering Co., Ltd., Nanjing, China) according to the manufacturer’s instructions.

### Real-time quantitative PCR analysis of mRNA levels

RNA was extracted from liver tissue using TRIzol Reagent® (Invitrogen, Shanghai, China) for qRT-PCR analysis. Reverse transcription was performed according to the PrimeScript™ RT Reagent Kit (Takara, Japan) instructions to obtain cDNA. Duplicate qRT-PCRs were performed with SYBRGreen® qPCR Premix (Yinuoweizhen Technology Co., Ltd., Hunan, China) in a Light Cycler 480 Real-Time PCR System (Roche Applied Science, Penzberg, Germany). The qRT-PCR mix consisted of 2 μL cDNA, 0.8 μL forward and reverse primer, 6.4 μL dH_2_O and 10 μL Taq SYBR® Green qPCR PreMix. qRT-PCR analysis was performed by the comparative CT method. The primers were produced by Sangon Biotech (Shanghai, China), and the primer sequences are listed in Table 1.

**Table 1:** Sequences of primers used in real-time PCR

Gene	Forward primer (5′- > 3′)	Reverse primer (5′- > 3′)
HGF	TGATCAACTCAGACGGCCTA	AGCCCCAGCACATATTTCAG
CyclinD1	AAGTGCGTGCAGAAGGAAAT	AGGAAGCGGTCCAGGTAGTT
TGF-β	CCATTCGCGGCCAGATT	GCTCCGGTTCGACACTTTC
β-actin	TCTGGCACCACACCTTCT	TGATCTGGGTCATCTTCTCAC

### Western blot analysis

Liver tissues were homogenized using Tissue Protein Extraction Reagent supplemented with 1 mM PMSF (Beyotime, Shanghai, China) in an automated fast sample grinder (Jingxin, Shanghai, China). Equal amounts of protein (30 μg) were resolved by sodium dodecyl sulphate-polyacrylamide gel electrophoresis (SDS-PAGE) and then electroblotted onto a nitrocellulose filter membrane (Pall Corporation, USA). The membrane was blocked in TBST containing 5% nonfat powdered milk for 2 h at room temperature to prevent nonspecific binding, incubated with primary antibody overnight at 4 °C, and then incubated with a horseradish peroxidase (HRP)-conjugated anti-species secondary antibody for 2 h. Antibodies against PCNA (Abcam, Cambridge, UK), p-STAT3, STAT3, suppressor of cytokine signalling 3 (SOCS3) (ImmunoWay, Plano, USA) and β-actin and a horseradish peroxidase (HRP)-conjugated antibody (Sangon Biotech, Shanghai, China) were used. Protein bands were visualized using Hypersensitive ECL Reagent (Meilunbio®, Dalian, China) and analysed using the Tanon 5200 Imaging System (Tanon Science & Technology Co., Ltd., Shanghai, China).

### Immunohistochemical analysis of Ki67 expression

Fresh liver tissue samples were fixed in 4% paraformaldehyde for 24 h and then embedded in paraffin using standard methods. Serial sections (5 μm thick) were dewaxed and rehydrated. The tissue sections were immersed in a 3% hydrogen peroxide solution to eliminate endogenous peroxidase, 0.1 M citrate buffer was used for antigen repair, and then the sections were blocked with BSA. Following incubation with the primary antibody (Novus, USA) overnight at 4 °C, the sections were washed with PBS and then incubated with a HRP-conjugated goat anti-rabbit secondary antibody (Boster, Wuhan, China) at room temperature for 30 min. Then, DAB staining was performed, and the sections were counterstained with haematoxylin. All sections were observed by light microscopy and quantified with Image-Pro Plus 6.0 software (Media Cybernetics, USA).

### Enzyme-linked immunosorbent assay (ELISA) of CM from ASCs

The method used to produce the CM was described above. After cell starvation, five bottles of cells were collected as parallel samples, and the supernatant was collected under aseptic conditions. ELISA was used to detect and evaluate cytokines of ASC-CM in this study. Following the manufacturer’s instructions, kits from Nanjing Jiancheng Bioengineering Co., Ltd. (Nanjing, China) were used to detect the concentrations of the following factors in CM: IL-6, IL-1β, TNF-α, ANG-1, ANG-2, VEGF and basic fibroblast growth factor (b-FGF).

### Statistical analysis

All data were analysed using GraphPad Prism 7.04 (GraphPad Software, USA) and are presented as the mean ± standard deviation. Statistical significance was determined by one-way ANOVA. A *p* value less than 0.05 was considered statistically significant.

## Results

### Identification of ASCs characteristics

Fourth-generation ASCs showed the characteristics of MSCs: they expressed the MSC markers CD90, CD29 and CD44 but did not express the haematopoietic marker CD34. The ASCs successfully differentiated into adipocytes, osteoblasts and hepatocytes in suitable media. We have previously described the isolation, culture and identification of ASCs [21].

### ASC-secretome improves histopathology after liver injury

**Fig. 1:**
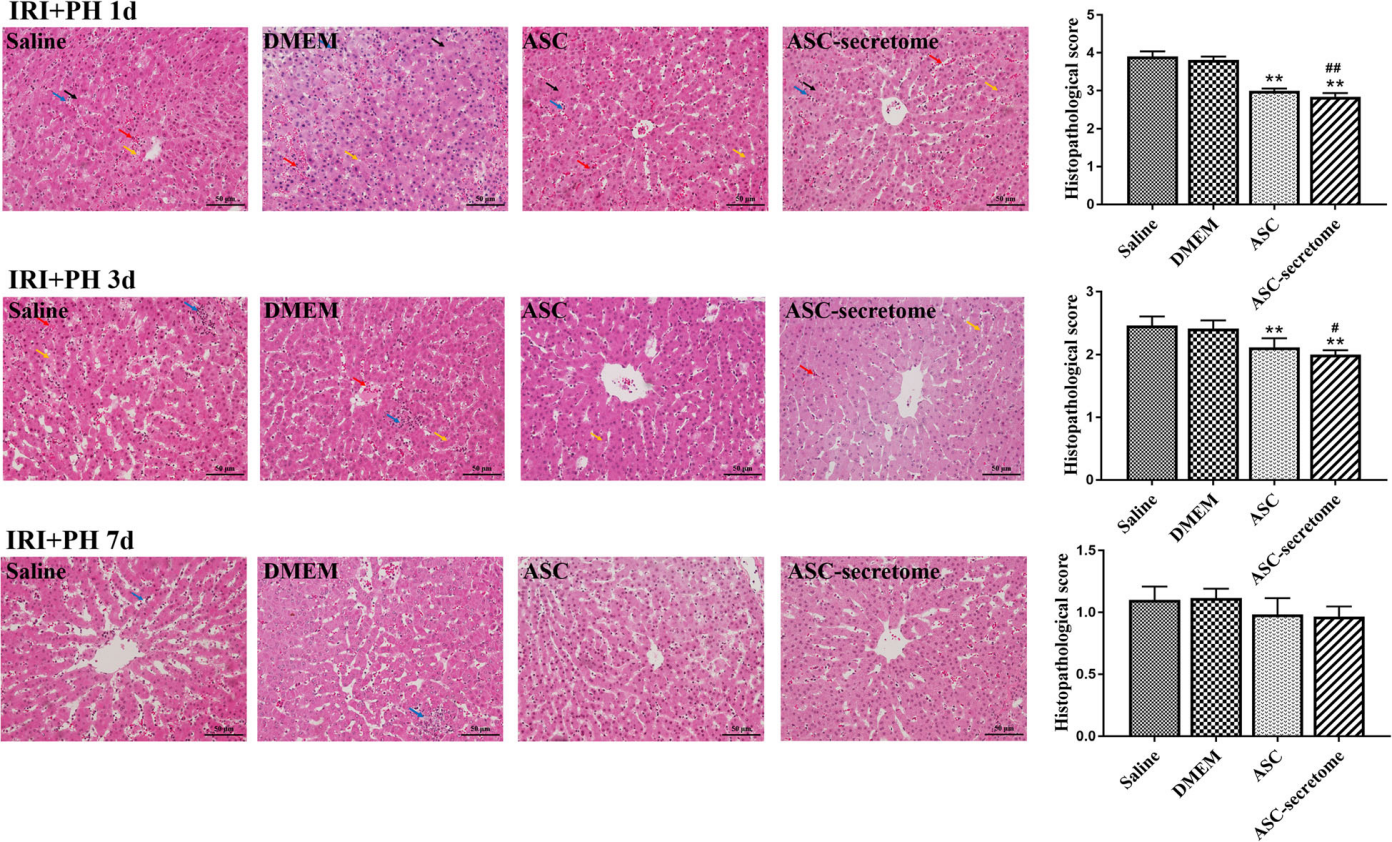
Histopathological changes and results of histological analysis results (original magnification × 400). The red arrow indicates haemorrhage, the yellow arrow indicates hepatocyte vacuolar degeneration, the black arrow indicates hepatocyte necrosis, and the blue arrow indicates inflammatory cells. The data are expressed as the mean ± SD. ∗*p* < 0.05, ∗∗*p* < 0.01, versus the saline group. ^#^*p* < 0.05, ^##^*p* < 0.01, versus the DMEM group.

Liver tissue pathological sections and the results of histological analysis are shown in Fig. 1. Sections from each group were observed under a microscope and scored histologically according to the Suzuki classification system. On the 1st postoperative day, obvious vacuolar degeneration and hepatocyte swelling were observed in the saline group and the DMEM group; additionally, the cord-like arrangement of hepatocytes was disrupted, and a large number of inflammatory cells were observed. In contrast, hepatocytes in the ASC group and the ASC-secretome group were slightly swollen and less vacuolated and had fewer inflammatory cells. The pathological scores showed that the difference between the treated group and the untreated group was extremely significant (*p* < 0.01), and the difference between the ASC-secretome group and the DMEM group was significant (*p* < 0.05). Three days after surgery, multiple focal necroses and more inflammatory cell infiltration were seen in the saline group and the DMEM group, while a small amount of inflammatory cell infiltration was seen in the ASC group and the ASC-secretome group, and no necrotic foci or vacuolar degeneration of liver cells was found. Additionally, hepatocyte swelling was milder than that of the untreated group. The pathological scores showed that the difference between the treated group and the untreated group was extremely significant (*p* < 0.01), and the difference between the ASC-secretome group and the DMEM group was significant (*p* < 0.05). At 7 days postoperatively, only a few inflammatory cells were seen in the saline group and the DMEM group, while there were almost no inflammatory cells in the visual field of the ASC group and the ASC-secretome group. The cord-like arrangement of hepatocytes was basically restored, and hepatocyte swelling disappeared. The pathological scores showed no significant differences between the groups.

### Detection of cytokines and growth factors in ASC-secretome

We detected the cytokines and growth factors in the CM of fourth-generation ASCs. ELISA was used to detect the concentrations of cytokines and growth factors in CM after 48 h of serum-free starvation. The concentrations of TNF-α, IL-6, IL-1β, ANG-1, ANG-2, VEGF, and b-FGF were 47.78 ± 3.2 pg/ml, 255.12 ± 16.97 pg/ml, 183.13 ± 7.49 pg/ml, 957.7 ± 90.79 pg/ml, 5.81 ± 0.23 ng/ml, 75.31 ± 4.62 pg/ml and 305.23 ± 23.18 pg/ml, respectively.

### ASC-secretome reduces inflammation after liver injury

**Fig. 2:**
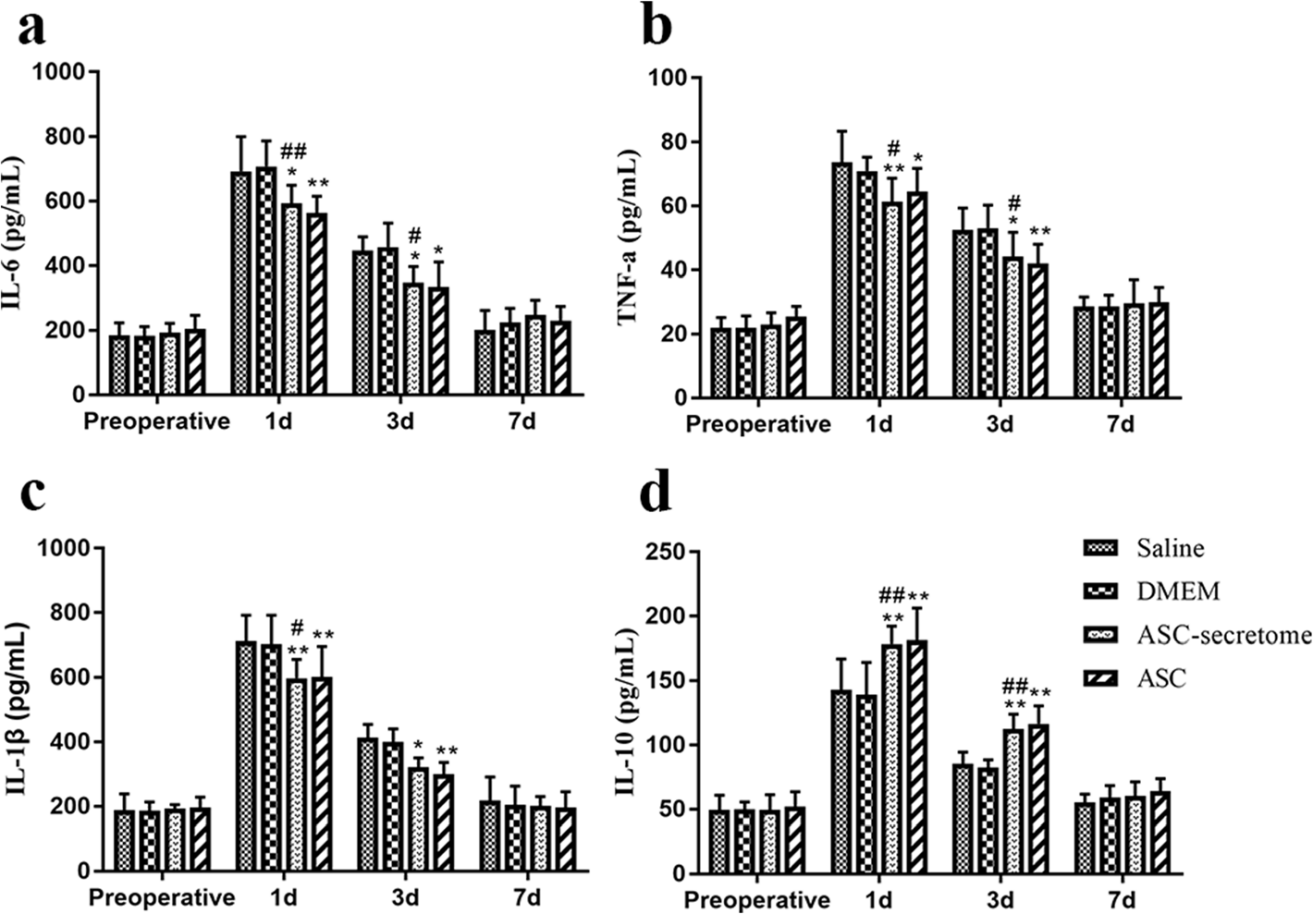
Effects of ASCs and the ASC-secretome on the serum levels of inflammatory response indicators. The serum levels of the inflammation indicators IL-6 (**a**), TNF-α (**b**), IL-1β (**c**) and IL-10 (**d**) are shown. The data are expressed as the mean ± SD. ∗*p* < 0.05, ∗∗*p* < 0.01, versus the saline group. ^#^*p* < 0.05, ^##^*p* < 0.01, versus the DMEM group.

The serum inflammation indicator results are shown in Fig. 2. Compared with those in the saline group, the serum TNF-α and IL-1β levels in the ASC-secretome group were significantly decreased 1 day after surgery (*p* < 0.01) and 3 days after surgery (*p* < 0.05), and the serum IL-6 levels were significantly different at 1 and 3 days after surgery (*p* < 0.05). The serum IL-6 levels of the ASC-secretome group and the DMEM group were significantly different at 1 day (*p* < 0.01) and 3 days (*p* < 0.05) after surgery (Fig. 2a), and the serum TNF-α and IL-1β levels of the ASC-secretome group were significantly different 1 day after surgery (*p* < 0.05, Fig. 2b, c). The serum IL-10 levels showed an overall upward trend after the operation, and the increase in the treatment group was greater than that in the control group (Fig. 2d). Both the ASC-secretome group and the ASC group had significantly increased serum IL-10 levels at 1 and 3 days after surgery (*p* < 0.01). Compared with those in the DMEM group, the levels of serum IL-10 in the ASC-secretome group were significantly different at 1 and 3 days after surgery (*p* < 0.01).

### ASC-secretome promotes liver regeneration after liver injury

**Fig. 3:**
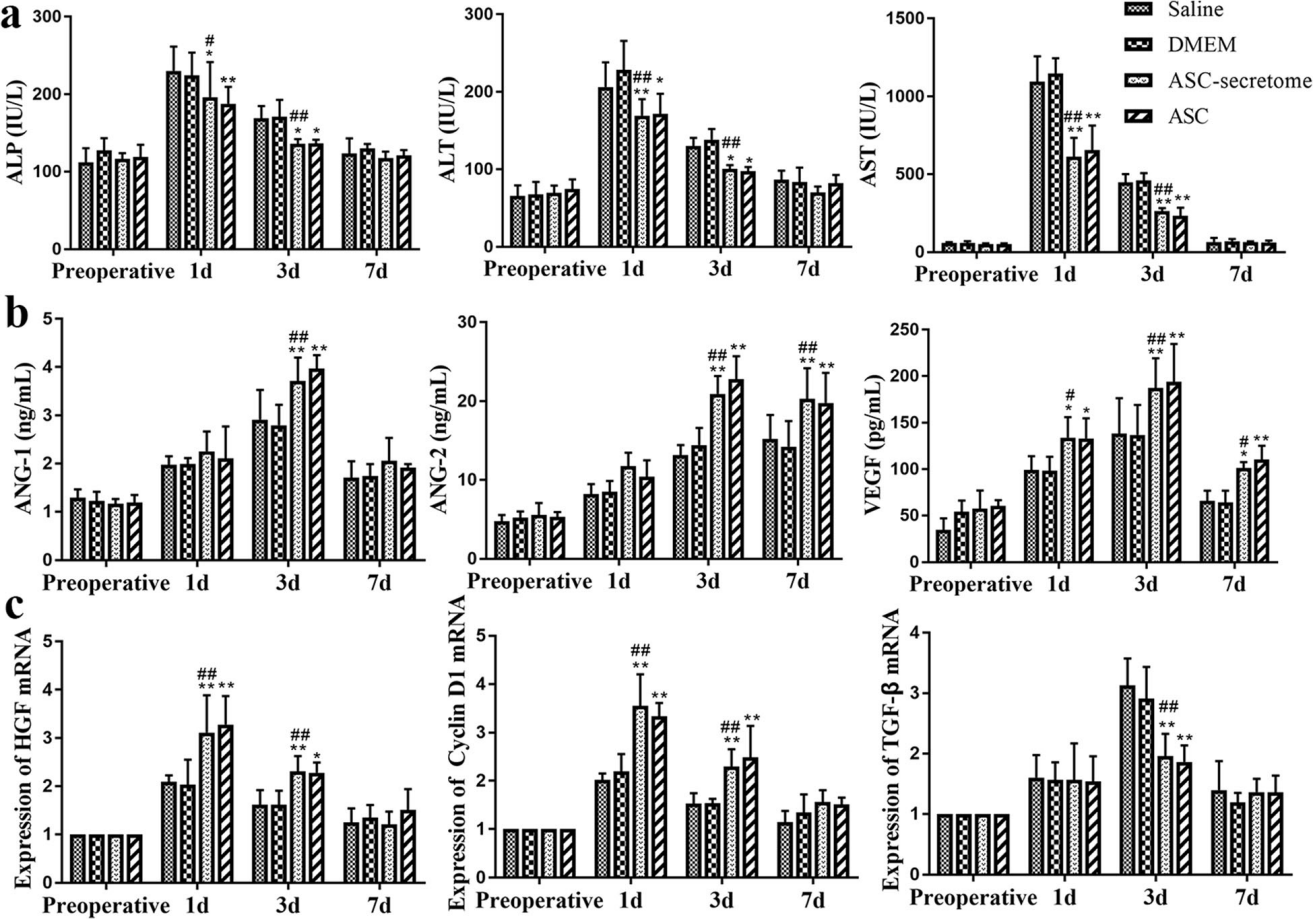
Effects of ASCs and the ASC-secretome on liver function and regeneration-related growth factors and genes. The liver functions of ALP, ALT and AST are shown in **a**. The angiogenesis-related factors ANG-1, ANG-2 and VEGF are shown in **b**. The liver regeneration-related genes HGF, Cyclin D1 and TGF-β are shown in **c**. The data are expressed as the mean ± SD. ∗*p* < 0.05, ∗∗*p* < 0.01, versus the saline group. ^#^*p* < 0.05, ^##^*p* < 0.01, versus the DMEM group.

Serum ALP, ALT and AST levels were measured to evaluate the damage to hepatocytes after ischaemia-reperfusion combined with partial resection. As shown in Fig. 3a, the concentrations of ALP, ALT and AST in the serum increased rapidly after surgery and then decreased gradually over time. The levels of ALP in the serum of the ASC-secretome group were significantly reduced at 1 and 3 days after surgery (*p* < 0.05) compared with those of the saline group, and the ALP levels in the ASC-secretome group were also significantly lower than those in the DMEM group (1 day: *p* < 0.05; 3 days: *p* < 0.01). Compared with the saline group, the ASC-secretome group had significantly reduced serum ALT levels after surgery (1 day: *p* < 0.01; 3 days: *p* < 0.05), while the ASC-secretome group also had significant differences compared with the DMEM group (*p* < 0.01). Compared with the control groups (saline and DMEM groups), the ASC-secretome group had significantly reduced levels of AST at 1 and 3 days after surgery (*p* < 0.01).

The serum levels of ANG-1, ANG-2 and VEGF increased significantly after surgery (Fig. 3b). Compared with the saline group, the ASC-secretome group and the ASC group had significantly increased serum VEGF levels 1 day after surgery (*p* < 0.05); 3 days after surgery, the ASC-secretome group and the ASC group had significantly increased serum levels of ANG-1, ANG-2 and VEGF (*p* < 0.01), and the difference in these factors between the ASC-secretome group and the DMEM group was highly statistically significant (*p* < 0.01). After 7 days, compared with the control group, the ASC-secretome group and the ASC group still had significantly increased expression levels of serum ANG-2 (*p* < 0.01). The treatments significantly promoted the expression of angiogenesis-related factors after liver ischaemia-reperfusion combined with partial resection.

After liver ischaemia-reperfusion combined with partial resection, the hepatocytes entered the proliferation phase, and the expression of hepatocyte growth factor increased (Fig. 3c). Compared with the saline group, the ASC-secretome group and the ASC group had significantly increased HGF and Cyclin D1 mRNA expression in liver tissue 1 day after surgery (*p* < 0.01). Three days after surgery, the treatment group still showed higher HGF and Cyclin D1 mRNA expression in liver tissue, but there was a significant inhibition of TGF-β mRNA expression in liver tissue (*p* < 0.01).

**Fig. 4:**
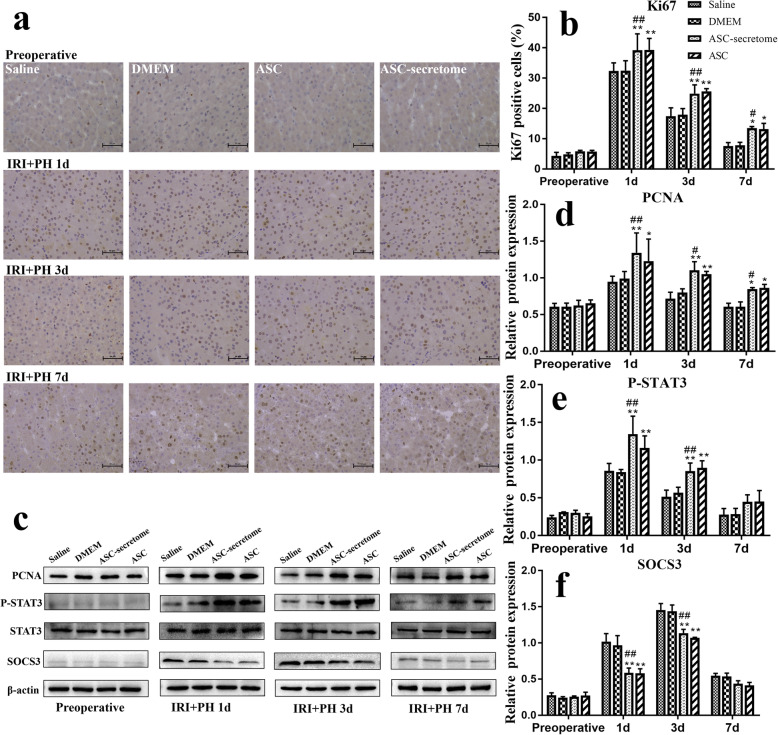
Effects of ASCs and the ASC-secretome on liver regeneration-related proteins. **a** Immunohistochemical staining of the Ki67 protein in liver tissue sections at all time points in the four groups. **b** Results of Ki67 immunohistochemical analysis. **c** Western blot of liver regeneration-related proteins. **d** Analysis of PCNA protein expression levels. Figure 4e: Analysis of p-STAT3 protein expression levels. **f** Analysis of SOCS3 protein expression levels. The data are expressed as the mean ± SD. ∗*p* < 0.05, ∗∗*p* < 0.01, versus the saline group. ^#^*p* < 0.05, ^##^*p* < 0.01, versus the DMEM group.

Figure 4a shows immunohistochemical staining of Ki67 in sections from each group at different time points. Analysis of the sections (Fig. 4b) indicated that compared with the control group, the ASC-secretome group and the ASC group had significantly increased the rates of Ki67-positive cells (1 and 3 days: *p* < 0.01; 7 days: *p* < 0.05). Figure 4c shows the Western blots of proteins related to liver tissue regeneration, and their relative expression levels normalized to that of β-actin are shown in Fig. 4d–f. Compared with the control group, the ASC-secretome group exhibited significantly increased expression of PCNA (1 and 3 days: *p* < 0.01; 7 days: *p* < 0.05), increased phosphorylation of p-STAT3 (1 day and 3 days: *p* < 0.01), and significantly inhibited expression of SOCS3 (1 day and 3 days: *p* < 0.01) to promote liver regeneration.

## Discussion

We hypothesized that the ASC-secretome could promote liver regeneration after hepatic ischaemia-reperfusion combined with partial hepatectomy. We used laparoscopic technology to establish a miniature pig liver ischaemia-reperfusion model with partial hepatectomy and performed liver parenchymal transplantation of the ASC-secretome. Our study showed that the ASC-secretome not only reduced inflammation and promoted cell proliferation in the model of liver ischaemia-reperfusion combined with partial hepatectomy, but also activated the STAT3 signalling pathway to promote liver regeneration. To our knowledge, this is the first study to describe the effect of the ASC-secretome on liver regeneration after liver ischaemia-reperfusion combined with partial resection in miniature pigs. However, this study also has its shortcomings. A minimally invasive laparoscopic technique was used to collect samples, and the animals were not slaughtered, so the ratio of liver weight to body weight was not calculated during liver regeneration.

Liver ischaemia-reperfusion and partial hepatectomy damage the liver parenchyma, and the remaining healthy liver tissue will also undergo pathophysiological changes due to ischaemia-reperfusion. The content of AST in the serum is very low under normal physiological conditions because it is mainly located in the mitochondria of liver cells, but after HIRI occurs, AST is released into the blood. ALT is mainly distributed in liver cells. The liver cell membrane structure is destroyed after HIRI, which disrupts cell integrity and causes ALT to be released into the blood. After HIRI, liver cells produce excessive amounts of ALP, which enters the blood through the lymphatic tract and hepatic sinusoids, causing the ALP content in the blood to rise. ALT, AST and ALP are indicators that can sensitively reflect the function of the liver. In this study, it was observed that the ASC and ASC-secretome groups had reduced expression levels of ALT, AST and ALP compared with those of the control group at 1 and 3 days after surgery, indicating that ASCs and the ASC-secretome had obvious therapeutic effects. The ASC-secretome group was significantly different from the DMEM group, and there were no significant differences between the DMEM group and the saline group, indicating that the basal medium had no therapeutic effect, while the ASC-secretome had a therapeutic effect due to the effective biological components secreted by ASCs into the basal medium. Similar results have been obtained in other studies. Majid Lotfini [22] used 15-fold concentrated MSC-CM to treat thioacetamide-induced acute liver failure in mice and observed significantly reduced expression levels of ALT and AST. Sang [23] reported that ASC-CM reduced liver damage and improved the liver microenvironment after ischaemia-reperfusion injury.

HIRI involves inflammatory pathways triggered by multiple inflammatory factors. It causes the secretion of a large number of pro-inflammatory factors to stimulate excessive inflammation, and the expression of anti-inflammatory factor IL-10 also increases accordingly. IL-10 can inhibit the release of inflammatory mediators such as TNF-α, IL-1β and IL-6 from mononuclear macrophages. Studies have shown that the CM of bone marrow mesenchymal stem cells (BMSCs) reduced the serum concentrations of TNF-α, IL-1β and IL-6; in contrast, the level of the anti-inflammatory cytokine IL-10 significantly increased in rats with radiation-induced liver injury [24]. Xu et al. [25] found that the ASC-secretome ameliorated neuroinflammation after traumatic brain injury and the IL-6 and TNF-α levels were reduced. Systemic infusion of MSC-CM also significantly reduced the expression levels of TNF-α and IL-6 in a rat model of acute liver injury induced by D-galactosamine [9]. The results obtained in this study are consistent with previous research. These findings revealed the effect of the ASC-secretome on the inflammatory response in liver ischaemia-reperfusion combined with partial hepatectomy injury.

The process of liver tissue regeneration and repair involves the activation, proliferation, and migration of vascular endothelial cells and the construction of new vascular networks. In this process, VEGF is an important paracrine factor of MSCs that promotes vascular endothelial cell proliferation and new blood vessel formation. ANG-1 stimulates blood vessel sprouting; ANG-2 plays an important role in angiogenesis by interacting with VEGF [26]. Gaetani et al. [27] proved that MSC-CM derived from the skin can promote the formation of capillaries. Among the cytokines involved in liver regeneration, HGF, the strongest known promoter of liver regeneration and its receptor c-Met, participate in the initiation of liver regeneration and play an important regulatory role in the process of liver cell proliferation [28, 29]. Cyclin D1 promotes the proliferation of hepatocytes. However, some evidence indicates that TGF-β plays a negative regulatory role in cell proliferation and prevents excessive cell proliferation [30]. In this study, the results showed that ASCs and the ASC-secretome significantly increased the levels of HGF and Cyclin D1 mRNA while inhibiting the increase in TGF-β mRNA. Similarly, Fouraschen et al. [31] reported that MSC-CM significantly increased the expression of HGF mRNA in liver tissue after 70% partial hepatectomy in mice, and Sang Chul et al. [32] found that the ASC-secretome significantly increased the expression of HGF protein and promoted liver regeneration.

**Fig. 5:**
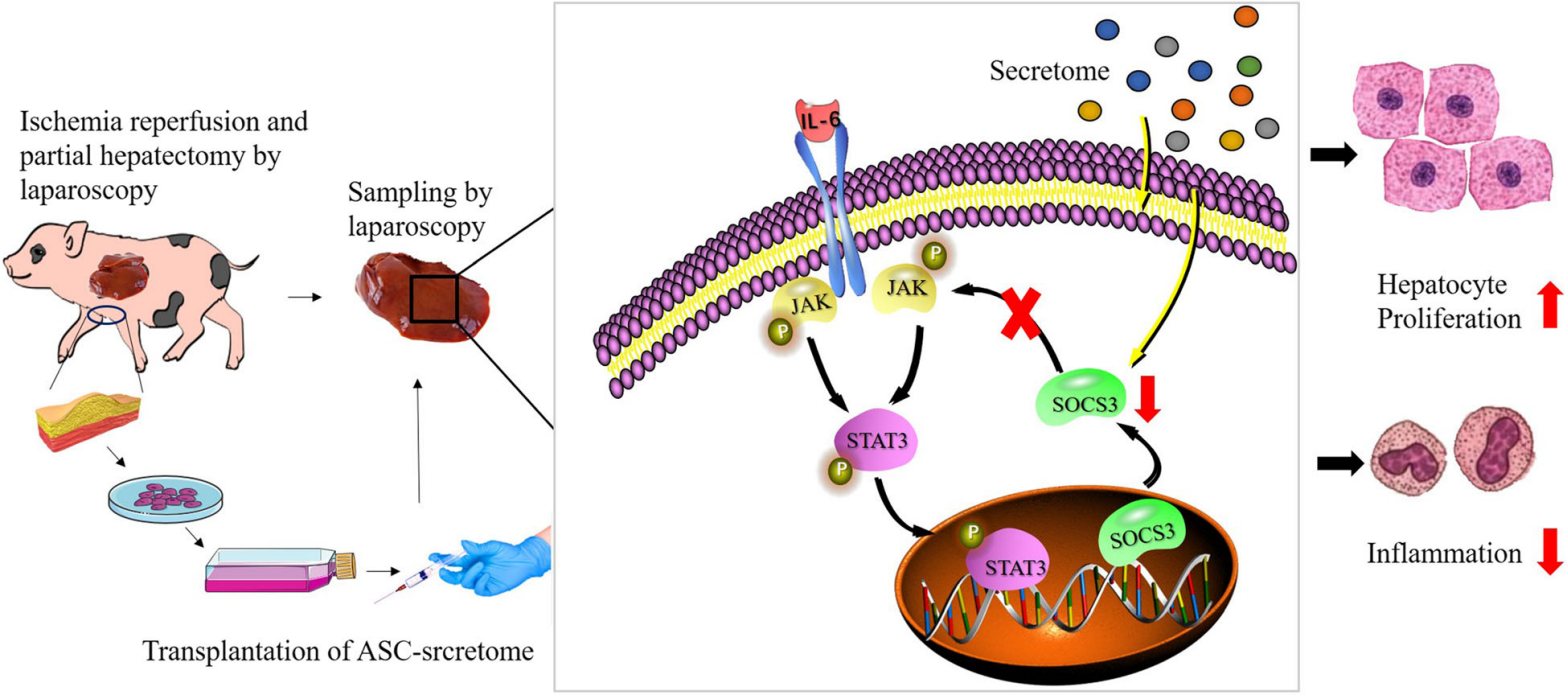
The experimental procedure and the mechanism by which the ASC-secretome promotes liver regeneration. The model of liver ischaemia-reperfusion combined with partial hepatectomy was established by laparoscopy. The ASC-secretome of miniature pigs was prepared and injected into the liver parenchyma immediately after the operation. The ASC-secretome exerted obvious anti-inflammatory and liver regenerative effects. The mechanism may be as shown in Fig. 5. After partial hepatectomy, the STAT3 signalling pathway was activated to promote the proliferation of hepatocytes. Furthermore, a SOCS3 negative feedback loop was activated to terminate the STAT3 signalling cascade. The ASC-secretome acted on SOCS3 to reduce its expression and interrupted the negative feedback regulation of the STAT3 signalling pathway, accelerating the process of liver regeneration. Cytokines in the ASC-secretome regulate inflammation and promote liver regeneration.

Hepatectomy caused an increase in the expression of inflammatory cytokines, among which IL-6 plays a prominent role in initiating liver regeneration. IL-6 can activate the downstream inflammation-related transcription factor STAT3 after binding to its receptor IL-6R to promote liver regeneration. Moreover, IL-6 increases the expression of SOCS3, which is an important target gene of STAT3, inhibits the phosphorylation of STAT3 and negatively regulates the process of liver regeneration. Studies have found that the IL-6/STAT3 pathway mediates cell proliferation and that the stem cell secretome obtained from hypoxia preconditioning promotes liver regeneration by inducing continuous expression of STAT3 in the liver, which is due to the decreased expression of SOCS3 [33]. In the study of Sang Chul et al. [32], the ASC-secretome significantly reduced serum IL-6 and TNF-α levels in partially hepatectomized mice, while the number of Ki67-positive cells increased significantly, and the expression of IL-6/STAT3 signalling pathway components reached its maximum. In this study, the results showed that the phosphorylation level of STAT3 increased and the expression of SOCS3 decreased compared with those in the control group. It is possible that the secretome inhibits the expression of SOCS3 through the action of cytokines in the liver microenvironment, delays negative feedback regulation of liver regeneration, reduces the expression of inflammatory factors, promotes hepatocyte proliferation and accelerates the process of liver regeneration (Fig. 5). PCNA is an important marker of liver regeneration. It is a cofactor of DNA polymerase that regulates DNA replication and the cell cycle. The Ki67 protein is expressed in proliferating cells. Therefore, PCNA and Ki67 protein levels can reflect the proliferation status of liver cells [34]. In a study of ASC-secretome treatment of liver resection, the expression of PCNA in the ASC group and ASC-secretome group were higher than that in the control group, and the phosphorylation levels of STAT3 increased, which significantly promoted liver regeneration [17]. In other studies, BMSCs and BMSC-CM were shown to inhibit necrotizing inflammation, increase Ki67 expression and promote liver regeneration [35]. MSC-CM increased the protein expression of Ki67 after 50% OLT in rats and promoted the proliferation of liver parenchymal cells [36]. Similarly, the present study showed that both ASCs and the ASC-secretome promoted the protein expression of PCNA and Ki67, which is consistent with the above results.

In this study, we also compared the effects of injection of ASCs and the ASC-secretome on liver regeneration. MSC-CM contains a variety of cytokines, chemokines and growth factors that have multiple positive effects on wound healing by improving endothelial cell migration and angiogenesis [37]. Studies have shown that cytokines do not work alone. For example, when VEGF and b-FGF are used in combination, they effectively promote the formation of collateral vessels in a rabbit hindlimb ischaemia model [38]. Various cytokines in the ASC-secretome work together to promote the repair of liver damage, reduce inflammation and promote liver regeneration. Our study showed that the ASC-secretome had the same promoting effect on liver regeneration as ASCs. Therefore, this study proved the potential of using the ASC-secretome to promote liver regeneration after liver ischaemia-reperfusion combined with partial resection.

## Conclusions

Our study highlights the potential of using the ASC-secretome to promote liver regeneration after liver ischaemia-reperfusion combined with partial resection. Importantly, we concluded that in a mammalian model organism, the miniature pig, the ASC-secretome exerted the same effect as ASCs transplantation. In particular, the ASC-secretome showed anti-inflammatory, pro-angiogenic and liver regenerative effects in this study. Considering the disadvantages of stem cell transplantation, the current study demonstrates the ASC-secretome as an advanced alternative to stem cell therapy for liver regeneration.

## Data Availability

The datasets used and/or analysed during the current study are available from the corresponding author on a reasonable request.

## Abbreviations

HIRI: Hepatic ischaemia-reperfusion injury
CM: Conditioned medium
ASC: Adipose-derived mesenchymal stem cell
ASC-secretome: Adipose-derived mesenchymal stem cell secretome
BMSCs: Bone marrow mesenchymal stem cells
IL-6: Interleukin-6
TNF-α: Tumour necrosis factor alpha
IL-1β: Interleukin-1 β
IL-10: Interleukin-10
ALP: Alkaline phosphatase
ALT: Alanine aminotransferase
AST: Aspartate aminotransferase
ANG-1: Angiopoietin-1
ANG-2: Angiopoietin-2
VEGF: Vascular endothelial growth factor
HGF: Hepatocyte growth factor
TGF-β: Transforming growth factor-β
PCNA: Proliferating cell nuclear antigen
STAT: Signal transducer and activator of transcription
SOCS3: Suppressor of cytokine signalling 3
